# Laws and Regulations of the Medical Benevolent Society

**Published:** 1823-03-01

**Authors:** 


### LAWS

AND

### REGULATIONS

OF THE

### Medical Benevolent Society,

INSTITUTED IN THE YEAR 1816.

---

Patron,

HIS ROYAL HIGHNESS THE DUKE OF SUSSEX.

---

### *LIST OF OFFICERS.*

President,

JOHN LATHAM, M.D.

Vice-Presidents:

| | | |
|---|---|---|
| JOHN HULL, M.D. | HENRY CLINE, Esq. | A. TEGART, Esq. |
| Sir H. HALFORD, Bart. | Sir A. COOPER, Bart. | R. WALKER, Esq. |
| Sir M. TIERNEY, Bart. | JOHN ABERNETHY, Esq. | E. A. BRANDE, Esq. |

Directors:

| *Physicians.* | *Surgeons.* | *Apothecaries.* |
|---|---|---|
| T. COOKE, M.D. | W. NORRIS, Esq. | W. MALIM, Esq. |
| P. M. LATHAM, M.D. | G. F. LOCKLEY, Esq. | H. ROBINSON, Esq. |
| ROBERT BREE, M.D. | H. L. THOMAS, Esq. | E. BROWNE, Esq. |
| G. M. BURROWS, M.D. | J. HAYES, Esq. | NEVILLE WELLS, Esq. |
| G. G. CURREY, M.D. | MARTIN WARE, Esq. | R. SIMMONS, Esq. |
| T. TURNER, M.D. | E. STANLEY, Esq. | JOHN HUNTER, Esq. |

Trustees:

| | |
|---|---|
| JOHN LATHAM, M.D. | JOHN BAYFORD, Esq. |
| HENRY CLINE, Esq. | RICHARD OGLE, Esq. |

Treasurers:

R. CLUTTERBUCK, M.D. New Bridge-street.
HENRY FIELD, Esq. Christ's Hospital.
RICHARD OGLE, Esq. Great Russell-street, Bloomsbury.

*Secretary,*—Mr. H. C. FIELD, 95, Newgate-street.
*Solicitor,*—CHARLES MURRAY, Esq. 13, John-street, Bedford-row.
*Collector,*—Mr. THOMAS UPTON, Cheltenham Depot, Throgmorton-street.
*Bankers,*—Messrs. CHILDS, Temple Bar.

---

*Subscriptions and Benefactions are received by the Treasurers, the Secretary, the Collector, and the Bankers.*

### PREFACE.

THE MEDICAL BENEVOLENT SOCIETY *is founded for the relief of those Members who are in distressed circumstances from mental or bodily infirmity, or who, from other causes, shall be considered as requiring and deserving of assistance, (provided they shall have been Subscribers for ten years); for which purpose a Fund has been formed.*

*When the objects of the Society are well understood, it cannot be doubted but that the less affluent of the Medical Profession will feel it prudent to appropriate a trifle from their annual incomes, to secure to themselves an addition to their comforts when age or infirmities shall have overtaken them; and that those also, who feel themselves happily raised above such contingencies, will not hesitate to contribute towards alleviating those casual misfortunes which too frequently oppress their less fortunate Brethren.*

### LAWS

OF THE

### Medical Benevolent Society.

*Objects of the Society.*

THE objects of the Society, and for which a Fund has been formed, are to afford relief to those Members who are in distressed circumstances, from mental or bodily infirmity, or who from other causes shall be considered as requiring and deserving of assistance.

### I. *Qualification and Admission of Members.*

1.—Any Physician, Surgeon, or Apothecary, residing and regularly practising his profession within England or Wales, is eligible to the Society.

2. Every person desirous of becoming a Member, must be recommended by at least one Member of the Society, who shall signify in writing to the Court of Directors, his name, line of practice, place of abode, and general character. He shall then be proposed at the Court of Directors, and balloted for at the next Court. And in every summons calling a Court of Directors, the names and residences of the persons to be balloted for shall be expressed. The form of the recommendation shall be as follows:—

"We, whose names are hereunto subscribed, do recommend A.B. [*here insert his line of profession, and place of abode*] believing him to be a gentleman of good character, and proper to become a Member of this Society.

*Dated,* Signed { C. D. E. F."

3. Any person proposed as a Member by the Chairman of a General Court, may be immediately balloted for, and elected by the same General Court.

4. Every person who shall be elected a Member of the Society, shall, on his admission, pay the sum of One Guinea to the Society, and shall also become a Subscriber to the Fund.

5. If any Member relinquish the practice of Medicine, he shall, nevertheless, continue a Member, and be entitled to all the privileges of the Society.

### PREFACE.

THE MEDICAL BENEVOLENT SOCIETY *is founded for the relief of those Members who are in distressed circumstances from mental or bodily infirmity, or who, from other causes, shall be considered as requiring and deserving of assistance, (provided they shall have been Subscribers for ten years); for which purpose a Fund has been formed.*

*When the objects of the Society are well understood, it cannot be doubted but that the less affluent of the Medical Profession will feel it prudent to appropriate a trifle from their annual incomes, to secure to themselves an addition to their comforts when age or infirmities shall have overtaken them; and that those also, who feel themselves happily raised above such contingencies, will not hesitate to contribute towards alleviating those casual misfortunes which too frequently oppress their less fortunate Brethren.*

### LAWS

OF THE

### Medical Benevolent Society.

---

### *Objects of the Society.*

THE objects of the Society, and for which a Fund has been formed, are to afford relief to those Members who are in distressed circumstances, from mental or bodily infirmity, or who from other causes shall be considered as requiring and deserving of assistance.

### I. *Qualification and Admission of Members.*

1.—Any Physician, Surgeon, or Apothecary, residing and regularly practising his profession within England or Wales, is eligible to the Society.

2. Every person desirous of becoming a Member, must be recommended by at least one Member of the Society, who shall signify in writing to the Court of Directors, his name, line of practice, place of abode, and general character. He shall then be proposed at the Court of Directors, and balloted for at the next Court. And in every summons calling a Court of Directors, the names and residences of the persons to be balloted for shall be expressed. The form of the recommendation shall be as follows:—

"We, whose names are hereunto subscribed, do recommend A.B. [*here insert his line of profession, and place of abode*] believing him to be a gentleman of good character, and proper to become a Member of this Society.

*Dated,* Signed { C. D. E. F."

3. Any person proposed as a Member by the Chairman of a General Court, may be immediately balloted for, and elected by the same General Court.

4. Every person who shall be elected a Member of the Society, shall, on his admission, pay the sum of One Guinea to the Society, and shall also become a Subscriber to the Fund.

5. If any Member relinquish the practice of Medicine, he shall, nevertheless, continue a Member, and be entitled to all the privileges of the Society.

### II.—*Honorary Members.*

The Society may admit, as Honorary Members, such gentlemen, not of the Medical Profession, as they may judge worthy of that distinction: but such Members shall have no vote in the appropriation of the funds of the Society.

### III.—*Disqualification of Members.*

1. Every Member subscribing to the Fund by annual payments, having omitted to make the same for one whole year, shall cease to be a Member of the Society.

2. If any Member shall publicly advertise any quack medicine, or empirically to cure any disease, the Court of Directors, having had due proof thereof, shall order the Secretary to deliver, or cause to be delivered to him, personally, a copy of the Laws of the Society, and also a written notice, setting forth, that unless he discontinue such practice within one month after the said notice shall have been delivered to him, he shall be subjected to removal from the Society; and if, after the expiration of the time allowed, the Court of Directors receive proof that the notice hath been duly served, and that the said Member hath not, in consequence thereof, discontinued such empirical practice, he shall cease, *ipso facto*, to be a Member of the Society, and also shall forfeit to the Society all the moneys which he may have paid.

### IV.—*General Courts.*

1. The General Courts of the Society shall superintend, regulate, and control all the affairs and concerns of the Society. A General Court of the Society shall be held in the month of April, in every year, and shall be called the Annual General Court; and the time and place of meeting shall be fixed by the Directors at their Quarterly Court in March.

2. At every Annual General Court, previously to all other business, the Minutes of the last Annual General Court, and of the Extraordinary General Courts, and of the Courts of Directors respectively, held since the last Annual General Court, shall be read, and then the proceedings of the Courts of Directors held since the last Annual General Court, shall, if thought proper, be discussed; and such resolutions entered into, in regard to the same, as shall be deemed expedient or proper: but no new business shall be introduced at an Annual General Court, unless recommended by a Resolution of the Court of Directors.

3. At every Annual General Court, the following Officers shall be elected out of the Members—a President, two or more Vice-Presidents, three Treasurers; and at the same Court, the President, Vice-Presidents, and Treasurers, for the preceding year, shall go out of office, but shall be immediately re-eligible.

4. In addition to the President, Vice-Presidents, and Treasurers, who shall be Directors *ex officio*, there shall always be eighteen Directors, *viz.* six Physicians, six Surgeons, and six Apothecaries. And, at the Annual General Court in April, six new Directors shall be elected out of the Members; and at the same Court, six Directors shall go out of office. And, for the purposes of ascertaining which of the said Directors shall, for the time being, go out, a distinct list of the said three classes shall always be kept by the Secretary; and the names of the Directors in each class shall be written one under the other; and as to the present Directors in each class, it shall be determined by lot in what order their names shall stand in the list; and as to all future Directors in each class, their names shall be inserted at the bottom of the list, in the order in which they shall be respectively elected; and the two Directors, whose names shall for the time being stand first in each of the said lists, shall be the Directors to go out; but no Director going out shall be re-eligible till he shall have been one year, at least, out of office.

5. If, between the respective times of holding the Annual General Court, the office of any one or more of the Directors, not being one of the two Directors standing at the head of each of the said three lists to be kept by the Secretary as aforesaid, shall be vacated by death, resignation, or otherwise, every such vacancy shall be filled up as soon as possible, by an Extraordinary General Court, or by the next Annual General Court, out of the Members of the Society; and every person elected to fill up any such vacancy, shall continue in office for the same period as the person whose vacancy he supplies would have continued in office, if the same had not occurred.

6. An Extraordinary General Court of the Society may be called at any time, by any eleven Members signing a requisition for the same, specifying the object for which such Extraordinary General Court is to be called; and such requisition, when signed, shall be sent to the Secretary.

7. No business shall be transacted at any General Court, unless nine Members be present; and if such number of Members shall not be assembled within one hour after the time fixed for holding the General Court, or if a sufficient number of Members shall be present, but from the pressure of business an adjournment shall be thought advisable, then and in either of the said cases the Members present, or a majority of them, shall have power to adjourn the General Court to some other day, within a fortnight from that time, and shall fix the time and place of meeting.

8. No other business shall be transacted at an Extraordinary General Court besides the business for which it shall have been called; and no other business shall be transacted at an adjourned General Court besides the business which ought to have been transacted at the Court from which such adjournment took place; and, seven days at least previously to the time fixed for holding a General Court, whether annual, adjourned, or extraordinary, a summons shall be sent by the Secretary to each Member residing in London, and within the range of the threepenny post; and the said General Court shall also be advertised in two or more of the London daily papers; and in the summonses and advertisements the time and place for holding the General Courts shall be specified, and, if the General Court for the time being called shall be an extraordinary one, the object for which the same shall be called shall also be specified in the summonses and advertisements.

9. No new law shall be made, nor any existing law repealed or altered, until approved of by one General Court, and confirmed by the next General Court, by a majority of at least three-fourths of the votes of the Members present and not declining to vote at each of the said Courts; and the substance of every new law, repeal, or alteration, shall be specified in the summons to be sent to each Member, except for the purpose of making new laws, or of repealing or altering any of the existing laws; and a majority of the votes of the Members present and not declining to vote at any General Court, shall be sufficient to decide all questions relating to the business transacted at that Court.

### V.—*Court of Directors.*

1. A Court of Directors shall be held at least four times in every year; *viz.* in the months of March, June, September, and December. And at every Court of Directors, the Minutes of the last Court of Directors shall first be read; the Reports of the Treasurer, Secretary, and Collector, of such sums of money as may have been received or paid by them on account of the Society since the last Court of Directors, shall also be read; and directions shall be given for the discharge of all just demands, and for investing in the public funds all such sums as can be spared from the current service of the Society; and candidates for admission shall be balloted for; and the recommendations of new candidates shall be read; and applications for relief shall be received and considered, and occasional assistance granted; and all other business of the Society transacted.

2. No business shall be transacted at any Court of Directors, unless five Directors be present.

3. The Court of Directors shall fix their place of meeting, and shall also fix the place where the General Courts of the Society shall meet. They may also provide a proper house or offices for carrying on the business of the Society.

4. The Court of Directors shall appoint the Secretary and Collector of the Society, and such other officers or servants as they may deem necessary, and dismiss them at pleasure, and appoint others in their stead.

5. The Court of Directors shall have power at any time to order an Extraordinary General Court of the Society to be called, and the same shall be called in the manner hereinbefore required.

6. The Court of Directors shall have power to make Bye-Rules and Regulations for their own government, and for the management of the business of the Society, and shall have power to hold Meetings by adjournment, and also to require the Secretary to call an Extraordinary Court of Directors; and any seven Directors shall also have power at any time, by writing under their hands, to require the Secretary to call an Extraordinary Court of Directors at the time appointed in the requisition. But no Extraordinary Court of Directors required to be called by any seven Directors as aforesaid, shall be held at a distance greater than fourteen days from the time when such requisition shall have been left with the Secretary; and every Extraordinary Court of Directors, which the Secretary shall be required to call as aforesaid, shall be called by the Secretary, by his sending at least five days before the time appointed for holding the same, a summons to each Director at his usual place of residence.

7. At the Quarterly Court of Directors in March shall be chosen a Committee of three Auditors, for auditing the accounts of the Society for the current year.

8. The Quarterly Court of Directors in June shall be held at Eight o'Clock in the evening, at such place as shall be from time to time appointed by the Court of Directors, and shall fix the day and hour when the next Quarterly Court of Directors shall be held; and ever afterwards every preceding Quarterly Court of Directors shall fix the time and place when the next Quarterly Court of Directors shall be held.

### VI.—*Secretary.*

1. The Secretary shall attend at all General Courts of the Society, and at all Courts of the Directors, and at any Committees which may be appointed either by the General Courts of the Society or by the Court of Directors, and shall enter into proper books the minutes of the proceedings at all those meetings; and he shall keep the accounts of the Society, and take care of all books and papers belonging to it; and he shall call all the General Courts of the Society, and all Extraordinary Courts of Directors in the manner herein-beforc required.

2. The Secretary shall give notice to the next Court of Directors of all applications made to the Society for occasional assistance; and whenever the Court of Directors shall have ordered any sum or sums of money to be paid by the Treasurer to applicants, or to those persons who are properly authorized to receive the same, the Secretary shall inform them, by letter, of the exact time and place when and where such payments will be made.

### VII.—*Collector.*

1. The Collector, on his first entering into office, shall give such security for the due performance and execution of his trust as the Court of Directors may deem requisite.

2. The Collector shall be allowed a commission of one shilling in the guinea on all the annual subscriptions and benefactions, and a commission of sixpence in the guinea on all life subscriptions, and on all subscriptions exceeding five guineas paid into his hands, legacies excepted.

3. The Collector shall wait upon each annual subscriber, residing within four miles from St. Paul's, for his yearly payment, within one month after it becomes due, and shall for the same purpose apply by letter to all other subscribers.

### VIII.—*Trustees and Treasurers.*

1. All the moneys of the Society which shall be placed in the parliamentary stocks or public funds, shall be invested in the names of four Trustees, who shall be appointed by the General Court of the Society; and whenever, in consequence of death or resignation, or from any other cause, a vacancy shall occur in the trust, such vacancy shall be filled up by the next General Court of the Society.

2. The Treasurers shall receive all the moneys of the Society, and pay all demands thereon, according to the regulations of the Society, and the orders of the Directors.

3. The Trustees, Treasurers, and Directors, shall be kept harmless and indemnified for what they shall lawfully do, in pursuance of the regulations, bye-rules, or orders of the Society, and in its service; and the stocks, funds, and moneys of the Society, shall in the first place, and at all times, be appropriated to the indemnification of the said officers, and to the payment of all costs, charges, and expences, incurred in conducting the concerns of the Society.

The said officers are to be liable and answerable for their own acts and deeds only, and not for the acts and deeds of each other.

### IX.—*Regulations of Meetings, Elections, Ballots, &c.*

1. At all General Courts of the Society, and at the Court of Directors, the chair shall be taken by the President, or, in case of his absence, by one of the Vice-Presidents, or in case, of the absence of the President and all the Vice-Presidents, by one of the Directors present.

2. All motions, either at a General Court of the Society, or at the Court of Directors, shall be delivered to the Chairman in writing, and be read by him twice before any decision takes place thereon; and no Member shall be allowed to speak more than once on any motion, unless he first obtain leave from the Chairman for that purpose.

3. All elections shall be by ballot, and decided by a majority; but, in case of an equality of numbers, the Chairman shall have a second or casting vote.

4. Previously to the election of any person into any office, place, or trust of the Society, two or more Scrutators shall be appointed, who shall cast up the votes on every ballot, and report the numbers.

5. No General Court of the Society, or Court of Directors, or Committee, shall be considered as adjourned or broken up until the Secretary shall have read over the minutes of the transactions of that Court or Committee, as entered in the rough Minute Book, and until the Chairman shall have signed them before he leaves the Chair.

### X.—*Subjects of Relief.*

The Court of Directors shall, after the expiration of ten years from the establishment of the Society, but not before, have discretionary power to grant relief to any member of the Society under peculiar circumstances of distress, who shall have been at least ten years a Member. The amount of the relief to be afforded to such Member, to be according to the sound discretion of the Court of Directors: nor shall any person be entitled to relief who is not, or shall have ceased to be, a Member of the Society.

### XI.—*The Disposal of Money, and the Mode of applying for Relief.*

1. All moneys of the Society shall be immediately invested in Government securities, except so much as shall be necessary to answer the current charges of the Institution.

2. All applications from persons desirous of receiving relief from the Society must be laid before the Quarterly Court of Directors in June or December, before any such relief can be granted, except in cases of urgent distress. All persons, therefore, who are desirous of receiving relief from the Quarterly Court of Directors, which is to meet in March, must send their letters of application to the Secretary on or before the first day of December, that the said letter may be laid before the Court of Directors which meets in that month. And all persons desirous of receiving relief from the Quarterly Court in September, must send their letters of application to the Secretary on or before the first day of June, in order that the said letters may be laid before the Court of Directors which meets in that month.

3. The money to be given away by the Society shall be distributed by the Court of Directors half-yearly, at the Quarterly Court in March, and at the Quarterly Court in September; except in cases of urgent distress. The utmost amount of the sum to be thus distributed by the Quarterly Court in March, shall be determined at the Quarterly Court of Directors in the December preceding; and the utmost amount of the sum to be so distributed by the Quarterly Court in September, shall be determined at the Quarterly Court of Directors in the June preceding.

4. All moneys granted by the Court of Directors out of the Fund, shall be paid by the Treasurer to those persons who are properly authorized to receive the same, within twenty days after such moneys are granted; and the Secretary shall, by letter, inform such persons of the exact time and place when and where the said Treasurer will be ready to make such payments.

### XII.—*Anniversary Festival.*

A List of the Members and Benefactors, with a general Statement of the Accounts of the Society, shall be annually printed, and sent to each Member, immediately after the election of officers, and shall be laid before the Members at their Anniversary Festival, which shall be held in the month of May; and the Stewards shall be appointed out of the Members, by the Quarterly Court of Directors in December; and the time and place of such Festival, together with the mode of conducting it, shall be determined by the Stewards and the Directors conjointly.

### XIII.—*Subscribers to the Fund.*

I. Every Member of the Society, who shall on his admission subscribe the sum of £26 : 5*s*. to the Fund, in addition to his admission fine of One Guinea, shall be considered a Member for life; and every Member who shall on his admission subscribe the sum of One Guinea, in addition to his admission fine of One Guinea, shall be considered an annual Member, and shall continue to be considered an annual Member as long as he shall continue annually to subscribe the sum of One Guinea; and, after annually subscribing the sum of One Guinea for thirty successive years, shall be considered a life Member, and absolved from all future subscriptions.

2. Every Member who shall subscribe annually, shall be considered a life Member, and absolved from all future annual subscriptions, on his paying down to the Society, within seven years from the time he shall first have become a Subscriber, such a principal sum of money, as, together with the sums then already paid by him to the Society in annual subscriptions, will amount to the sum of £26 : 5*s*.

3. There shall be delivered to each Member a receipt for the amount of every subscription paid by him.

*Form of Letter of Application.*

To the President, Vice-Presidents, and Court of Directors, of the Medical Benevolent Society.

A. B. desires to inform the Court of Directors, that he is residing and practising as a              in the parish of              in the county of              that he is              years of age, and has been a Member of the Society since the year              that he is not in circumstances to support himself without the assistance of the Society.

(Signed)      A. B.

We whose names are hereunto undersigned, being well acquainted with the said A. B. do hereby certify, to the best of our knowledge and belief, that the contents of the above letter are true.

Dated, the      Day of      18      (Signed)      { C. D.

### LIST OF MEMBERS,

APRIL, 1822.

---

*Those marked thus † are Life Subscribers; thus * have served the office of Stewards at the Anniversary Festival.*

---

ABERNETHY, John, (S.) Bedford-row.
Acton, John, (S.) Ludlow.
Adams, Sir William, (S.) Albemarle-street.
Addison, James, (S.) Burnham, Essex.
* Ainslie, H., M.D. Dover-street.
Andrews, Richard, (S.) Stanmore, Middlesex.
Alexander, Henry, (S.) Cork-street.
Armstrong, John, M.D. Southampton-row, Bloomsbury.
Babington, W., M.D. Aldermanbury.
Backler, John, (A.)
† Bailie, Matthew, M.D. Lower Grosvenor-street.
Barrett, Robert, (A.) Walworth.
Bartley, Alfred Collett, (A.) Mitcham, Surrey.
Baylis, James, (A.) Thayer-street, Manchester-square.
Bellingham, James, (S.) Rye, Sussex.
Best, Henry, (A.) Thetford, Norfolk.
Best, Thomas, (S.) Tavistock-street.
Bliss, John, M.D. Bath, Somersetshire.
Blundell, James, M.D. St. Saviour's church-yard.
Bostock, ———, M.D. Upper Bedford-place.
Boulton, George, (S.) 17, Great Russell-street.
Boutflower, John Johnson, (A.) Richmond, Yorkshire.
Bowes, Christopher, (S.) Richmond, Yorkshire.
* Bowman, Michael, (A.) Harley-street.
* Brande, Everard, (A.) Arlington-street.
Briden, William, (S.) Great Coram-street.
Brown, Tobias, (S.) Camberwell.
* Browne, Edward, (S.) Raven-row, Spitalfields.
* Bree, Robert, M.D. George-street, Hanover-square.
Bryant, John, (A.) Edgeware-road.
Burchell, Benjamin, (A.) Pleasant-row, Pentonville.
* Burrows, George Man, M.D. Gower-street.
Cabbell, George, (A.) Chapel-street, Lisson-green.
Camplin, John Messendine, (S.) Finsbury-square.
† Cates, John, (A.) Argyle-street.
* Chambers, W. Frederick, M.D. Dover-street.
* Clarke, Charles Mansfield, (S.) Saville-row.
* † Cline, Henry, (S.) Lincoln's Inn-fields.
* Clutterbuck, Henry, M.D. New Bridge-street.
Coleman, Edward, (S.) Professor, Veterinary College.
Constable, Michael, (S.) Woodford, Essex.
* Cooke, John, M.D. Gower-street.
† Cooper, Sir Astley P., Bart. Spring-gardens.

Copeland, ———, (A.) Chigwell.
Copeland, Thomas, (S.) Golden-square.
* Cordell, W. D. (S.) Union-court, Broad-street.
* Currey, George Gilbert, M.D. Half-Moon-street.
Curtis, John, (S.) Lambeth-street, Goodman's-fields.
Dadley, Henry, (A.) Manchester.
Dallaway, M. (A.) Stratford, Essex.
Darling, George, M.D. Brunswick-square.
Davies, William, (A.) 13, Beckford-row, Walworth.
Dean, ———, (S.) Oxford-street.
Dickinson, Nodes, (S.) Wigmore-street, Cavendish-square.
Dickinson, William Benley, (S.) Macclesfield.
Downe, Nathaniel, (A.) Bridport, Dorsetshire.
† Drever, Thomas, M.D. Lower Grosvenor-street.
Drew, Walter, (S.) Gower-street.
Este, M. L. (S.) Hyde Park Barracks.
Ewbank, Andrew, (A.) Upper Grosvenor-street.
* Eyles, John Brown, (A.) St. Andrew's-court, Holborn.
Eyles, Richard Strong, (A.) St. Andrew's-court, Holborn.
Ferrick, Peter, (A.) Dorset-street, Manchester-square.
* Field, Henry, (A.) Christ's Hospital.
Field, Henry Cromwell, (S.) Newgate-street.
Field, Thomas, (A.) Wilderness-row, Goswell-street.
Fincham, George, (S.) Spring-gardens.
Fisher, John William, (S.) Greek-street, Soho.
Fiske, Charles, (S.) Saffron Walden, Essex.
Foote, William, (S.) Edgeware, Middlesex.
Francis, Charles, (A.) Bexley, Kent.
* † Gardner, Abraham, (S.) Clapham, Surrey.
Good, John Mason, M.D. Guildford-street.
Goolden, Richard, (S.) Maidenhead, Berks.
Graham, Thomas, (S.) Turnham-green.
Green, John Hornidge, (S.) Manchester-square.
Grice, George, (A.) Welbeck-street.
Griffith, Samuel, (A.) Tavistock-street, Bedford-square.
Gualter, H. (A.)
Guthrie, G. J. (S.) Berkeley-street.
Halford, Sir Henry, Bart. Curzon-street.
Hammerton, Thomas, (A.) Piccadilly.
Harkness, J. (S.) Broad-street, Ratcliffe.
* Hayes, Joseph, (S.) Upper Charlotte-street.
Haynes, John, (S.) Hampstead.
Higham, John, (A.) Lower Grosvenor-street.
Hill, John Wilkes, (S.) Cooper's-row.
Hillman, William, (S.) Argyle-street.
Hobson, George, (A.) Great Mary-le-bone-street.
Hodgshon, Richard, (A.) Darlington, Durham.
Hooper, Robert, M.D. Saville-row.
Hudson, George, (S.) Manchester.
Hull, John, M.D. Manchester.
Hunter, John, (A.) Tower-street.

Hunter, John, jun. (S.) Mincing-lane,
Hurlock, Joseph, (S.) St. Paul's Church-yard.
Ireland, Richard, (S.) Pendleton, Lancashire.
Irish, Thomas, T. (S.) Stamford-street.
* Jacob, Charles, (S.) Hampstead.
* James, Robert, (A.) Chapel-street, Bedford-row.
Jarvis, John, (A.) New Compton-street.
Johnson, George, (S.) John-street, America-square.
Johnson, J.N., M.D. Half Moon-street.
Jones, Jacob, (S.) Finsbury-square.
Jones, Thomas, (A.) Strand.
Jones, John, (A.) Mount-street, Grosvenor-square.
Jordan, Joseph, (A.) Manchester.
Keate, Robert, (S.) Albemarle-street.
Keats, Richard, (A.) Fulham, Middlesex.
Keele, Henry William, (S.) Cheyne-walk, Chelsea.
Kerswill, Samuel, (S.) Plymouth.
King, Richard Henry, (A.) Mortlake, Surrey.
King, William, (S.) George-street, Hanover-square.
Knighton, Sir William, Bart. M.D. Hanover-square.
Laird, James, M.D. Bloomsbury-square.
* † Latham, John, M.D. Harley-street, Cavendish-square.
* Latham, Peter Mere, M.D. Gower-street.
Lockley, George Frederick, (S.) Half Moon-street.
* Luke, Stephen, M.D. Argyle street.
Macdonald, William Rio, (A.) Doughty-street.
Mackinder, John, (S.) Middlesex-place, New-road.
Maiden, William, (S.) Stratford, Essex.
Malim, Wentworth, (A.) Lincoln's Inn-fields.
Martindale, James Ward, (S.)
* † Merriman, Samuel, M.D. Half Moon-street.
Miles, John, (A.) Throgmorton-street.
Monro, Edward Thomas, M.D. Gower-street.
Moore, Thomas, (A.) Norfolk-street.
Morrah, James, (S.) Sloane-street.
Newby, Charles, (A.) Poland-street.
Newnham, Richard, (S.) Brighton.
Newnham, William, (A.) Farnham.
Norris, William, (S.) Old Jewry.
* Nussey, John, (A.) St. James's-street.
Ogle, James Ady, M.D. Oxford.
Ogle, Richard, (S.) Great Russell-street, Bloomsbury.
Partington, Miles, (A.) Lower Brook-street.
* Parsloe, F.R. (S.) Upper Eaton-street, Pimlico.
† Pearson, John, (S.) Golden-square.
* † Pennington, R. R. (S.) Montague-place.
Peregrine, J.P. (S.) Half-Moon-street.
Philip, Wilson, M.D. Hanover-square.
Pidcock, John, M.D. Watford.
Plumbe, Samuel, (S.) Great Russell-street.
Pope, Jonas Hall, (A.) 6, Somerset-street, Portman-square.

Powel, John, (S.) Newman-street.
Price, Charles, M.D. Brighton.
Radford, Thomas, (S.) Manchester.
Ramsbotham, John, M.D. New Broad-street.
Ridout, John Gibbs, M.D. New Bridge-street.
Ridout, John, (S.) New Bridge-street.
* Robinson, Henry, (S.) Cooper's-row.
Robinson, William, (S.) Great Portland-street.
Rumsey, James, M.D. Amersham, Bucks.
Rumsey, James, Amersham, Bucks.
Sandford, W. M. (S.) Bridge-row, Lambeth.
Sawrey, Solomon, (S.) Bedford-row.
Scriven, Hugh, (A.) Great Marlborough-street.
* Seaton, James, (A.) Bridge-street, Westminster.
Sheldon, Ebenezer, (S.) Mount-street, Grosvenor-square.
Sherwin, John, M.D. Bath.
* Shillito, Charles, (S.) Putney.
Shuter, John Allen, (S.) Gainsford-street, Southwark.
Shuter, Charles, (S.) Guildford-street.
* Simmons, Richard, (S.) Oxendon-street.
Simmons, Robert, (S.) Oxendon-street.
Simons, William, (A.) Sydenham, Kent.
Smith, Southwood, M.D. Trinity-square.
Smith, Thomas, (A.) 63, Upper Berkeley-street, Portman-sq.
Southey, Henry Herbert, M.D. Queen-Ann-street.
* Stanley, Edward, (S.) Lincoln's Inn-fields.
Steele, Joseph, (S.) Trinity-square.
Stewart, John, (A.) Manchester.
Swift, James, (S.) Putney.
* † Tegart, Arthur, (A.) Pall Mall.
Tegart, Edward, (S.) Pall Mall.
* Thomas, H. Leigh, (S.) Leicester-place.
* Thomson, Antony Todd, (S.) Sloane-street.
Thompson, Thomas, M.D. Conduit-street.
Thorpe, Robert, (S.) Manchester.
* Tierney, Sir Matthew, Bart. M.D. Dover-street.
Turner, Henry, (S.) King-street, Bloomsbury.
* Turner, Thomas, M.D. Curzon-street.
Turner, William, (S.) New-street, Dorset-square.
Upton, James, (A.) Throgmorton-street.
Wadd, Thomas, (S.) Basinghall-street.
* Wadd, William, (S.) Park-place, St. James's.
Walker, John, M.D. Bond-court, Walbrook.
* Walker, Richard, (A.) St. James's-street.
Wallace, James, (A,) Bow.
Warburton, John, M.D. Clifford-street.
* † Ware, Martin, (S.) New Bridge-street.
† Ware, John, (S.) New Bridge-street.
Ward, Stephen Smith, (S.) Plaistow, Essex.
Waring, R. jun. (A.) Dover-street Piccadilly.
Washburn, N. (S.) Marlborough.

Watson, John, (S.) Berners-street.
Webbe, Thomas, (A.) Cold Bath-fields.
Welbank, John, (S.) Chancery-lane.
Wells, Richard Strong, (A.) St. Andrew's-court, Holborn.
Wells, Neville, (A.) Welbeck-street.
Wells, George, (A.) Welbeck-street.
Wheeler, Thomas, (A.) St. Bartholomew's Hospital.
* Williams, Reginald, (A.) Bloomsbury-square.
Wilson, William James, (S.) Manchester.
Winfield, William, (A.) 19, Buckingham-street, Strand.
Young, Samuel, (S.) Cancer Institution, Gerrard-street.

### *HONORARY MEMBERS.*

Right Honourable Earl of Rosslyn.
Right Honourable Earl of Blessington,
Most Noble Marquis of Lansdown.
Baker, Sir Frederick, Bart.
Forster, Edward, Esq. St. Helen's-place.
Doyle, Sir John, Bart. G. C. B.
Irish, Edward, Esq.
Murray, Charles, Esq. John-street, Bedford-row.
Warburton, Thomas, Esq. Hackney.
Thompson, Richard, Esq. Grosvenor-square.
Savory, Thomas Field, Esq. Bond-street.

### BENEFACTIONS.

| | £. | s. | d. |
|---|---|---|---|
| His Royal Highness the Duke of Sussex | 26 | 5 | 0 |
| Right Honourable Earl of Blessington | 21 | 0 | 0 |
| Sir Frederick Baker, Bart. | 105 | 0 | 0 |
| Edward Forster, Esq. | 10 | 10 | 0 |
| Edward Irish, Esq. | 1 | 1 | 0 |
| Charles Murray, Esq. (ann.) | 1 | 1 | 0 |
| Thomas Warburton, Esq. | 10 | 10 | 0 |
| Richard Thompson, Esq. | 50 | 0 | 0 |
| John Latham, M. D. President | 100 | 0 | 0 |
| H. Ainslie, M.D. | 10 | 10 | 0 |
| C. M. Clarke, Esq. | 10 | 10 | 0 |
| Henry Cline, Esq. | 50 | 0 | 0 |
| John Cooke, M.D. | 6 | 6 | 0 |
| Sir Gilbert Blane, Bart. | 10 | 10 | 0 |
| Dr. Bree | 10 | 10 | 0 |
| Sir Astley P. Cooper, Bart. | 50 | 0 | 0 |
| Thomas Graham, Esq. | 3 | 0 | 0 |
| John Hull, M.D. | 50 | 0 | 0 |
| Do. in addition to his Annual Subscription (ann.) | 1 | 1 | 0 |
| William Norris, Esq. | 10 | 0 | 0 |
| B. F. Outram, M.D. | 5 | 5 | 0 |
| John Abernethy, Esq. in addition to his Annual Subscription (annual) | 1 | 1 | 0 |
| John Walker, M.D. in addition to his Annual Subscription (annual) | 2 | 2 | 0 |
| The Society of Apothecaries (annual) | 5 | 5 | 0 |
| Murray Forbes, Esq. | 10 | 0 | 0 |
| Samuel Lawford, Esq. (ann.) | 2 | 2 | 0 |
| Stephen Smith Ward, Esq. | 5 | 5 | 0 |
| John Parrot, Esq. | 5 | 5 | 0 |
| George Johnson, Esq | 1 | 1 | 0 |
| John Stewart, Esq. Albany | 2 | 0 | 0 |
| Sir Matthew Tierney, Bart. | 10 | 10 | 0 |
| J. W. Duncan, Esq. | 2 | 2 | 0 |
| Thomas Field Savory, Esq. | 5 | 5 | 0 |
| Matthew Baillie, M.D. | 10 | 10 | 0 |
| Robert James, Esq. | 2 | 2 | 0 |
| William Lewis, Esq. | 5 | 0 | 0 |
| R. M. Kerrison, M.D. | 5 | 5 | 0 |
| Anonymous, by Dr. Baillie | 30 | 0 | 0 |
| Alfred Bartley, Esq. | 3 | 3 | 0 |
| Sir Henry Halford, Bart. | 20 | 0 | 0 |
| Sir Wm. Knighton, Bart. | 21 | 0 | 0 |

Printed by J. TWIGG, Richard Street, Islington.

